# Polymorphisms of *SLCO1B1* rs4149056 and *SLC22A1* rs2282143 are associated with responsiveness to acitretin in psoriasis patients

**DOI:** 10.1038/s41598-018-31352-2

**Published:** 2018-09-04

**Authors:** Wangqing Chen, Xu Zhang, Wei Zhang, Cong Peng, Wu Zhu, Xiang Chen

**Affiliations:** 1 Department of Clinical Pharmacology, Xiangya Hospital, Central South University, ChangSha, Hunan 410008 China; 2 Department of Dermatology, Xiangya Hospital, Central South University, Changsha, Hunan 410008 China; 3 Hunan Key Laboratory of Skin Cancer ans Psoriasis, ChangSha, Hunan 410008 China

## Abstract

Acitretin is widely used to treat psoriasis, but the efficacy varies significantly among individuals. To explore the association between polymorphisms and acitretin efficacy, we enrolled 46 and 105 Chinese Han psoriasis vulgaris patients for discovery and validation phases, respectively. The patients were treated with acitretin (30 mg/day) and calcipotriol ointment for at least 8 weeks, and their genotypes were detected. The wild-type genes and variants were transfected into HEK293 cells, which were then incubated with acitretin. The cellular acitretin concentration was measured by liquid chromatography-mass spectrometry. We found that the polymorphisms rs4149056 in the *SLCO1B1* gene and rs2282143 in the *SLC22A1* gene were associated with efficacy, both in the discovery (*P* = 0.013 and *P* = 0.002) and validation phases (*P* = 0.028 and *P* = 0.014), based on a 50% reduction from before to after treatment of the psoriasis area severity index (PASI50). When the PASI75 was used as an efficacy cutoff, a similar conclusion was drawn. The uptake of acitretin was lower with the rs4149056C (*P* = 0.002) and rs2282143T alleles (*P* = 0.038) than the wild-type alleles. Our results imply that the rs4149056C and rs2282143T variants decrease the acitretin uptake, and significantly associated with clinical effective responsiveness.

## Introduction

Psoriasis is a common stubborn inflammatory skin disease involving abnormal T-cell activation and inadequate keratinocyte differentiation, with lesions that are characterized by red, scaly patches, and itching^1^. Psoriasis can be accompanied by several serious complications such as dyslipidemia and metabolic disorders, and selecting a sustainable, long-term treatment for psoriasis is a challenge^2,3^.

Acitretin, a second-generation retinoid drug, is an aromatic synthetic derivative of all-trans-retinoic acid. Acitretin combines with the nuclear receptors retinoic acid receptor (RAR) and retinoid X receptor (RXR) and regulates transcription factors, such as signal transducer and activator of transcription 1 and nuclear factor-κB^4,5^. Acitretin induces keratinocyte differentiation and reduces epidermal hyperplasia, leading to slowing of cell reproduction^6^. Acitretin also exerts an important influence on the regulation of T-helper 1 and 17 cells^7^. Acitretin is widely used to treat moderate to severe psoriasis alone or in combination regimens, such as with calcipotriol. High doses (50–75 mg/day) may result in more rapid and possibly more complete responses, but high dosages are associated with significantly increased side effects. Thus, a low dosage (<50 mg/day) of acitretin is recommended^8–10^. Approximately, 23–52% of patients achieve a 75% improvement iof the psoriasis area severity index (PASI75) from baseline to after treatment, and 66–85% of patients show a 50% improvement of the PASI after 8–12 weeks of treatment^4,11^. Therefore, the efficacy of acitretin varies significantly among psoriasis patients. (Supplementary Figure S1).

Pharmacogenomics explores the links between genetics and drug responses. Genetic polymorphisms involving drug targets (i.e., pharmacodynamics), drug metabolism and transporters (i.e., pharmacokinetics) are likely to be important sources of individual variability in drug efficacy or adverse reactions^12,13^. Studies focusing on the metabolic processes of acitretin *in vivo* is few. One previous study suggested that retinoid drugs had some affinity to P-glycoprotein^14^, whereas the study by Holthoewer *et al*. found not^15^. Our previous studies showed that polymorphisms of *EGF* and *SFRP4* genes were associated with the response to acitretin in psoriasis^16–18^. Thus, the pharmacokinetics of acitretin *in vivo* is unclear. In this study, after screening the ADME chipset, polymorphisms of solute carrier organic anion transporter family member 1B1 (*SLCO1B1*) and solute carrier family 22 member 1 (*SLC22A1*) genes were found to be associated with the efficacy of acitretin.

The *SLCO1B1* and *SLC22A1* genes are mainly expressed in the liver. Several drugs have been shown to be substrates of these two transporters: pravastatin and repaglinide are transported by organic anion-transporting polypeptide 1B1 (OATP1B1, encoded by the *SLCO1B1* gene)^19,20^ and imatinib and metformin by organic cation transporter 1 (OCT1, encoded by the *SLC22A1* gene)^21^. The missense variant of rs4149056 (also known as c.521 T > C, Val174Ala, with the T allele defines as the wild-type allele and the C allele as a variant) is associated with reduced expression and activity of *SLCO1B1*^12^. The rs2282143 (c.1022 C > T, Pro341Leu, with the C allele defines as the wild-type allele and the T allele as a variant for rs2282143) polymorphism also affects the substrate uptake of OCT1, such as lamivudine and [^14^C]-tetraethylammonium^22,23^.

In this study, we suspected that the acitretin efficacy may vary due to pharmacogenetics and that polymorphisms of the *SLCO1B1* and *SLC22A1* genes may affect the uptake and efficacy of acitretin.

## Methods

### Patients

A total of 151 Chinese Han patients with moderate to severe psoriasis were enrolled into this study from August 2012 to September 2015. According to the reference, patients with a PASI score of more than 7 or a body surface area (BSA) of more than 10 were defined as having moderate to severe psoriasis;^24^ PASI = 0.1(Rh + Th + Sh)Ah + 0.2(Ru + Tu + Su)Au + 0.3(Rt + Tt + St)At + 0.4(Rl + Tl + Sl)Al, and BSA of 1 indicates that 1% of the body area is involved. Patients were treated with acitretin (Huapont Pharm., China) at 30 mg/d and calcipotriol ointment (Bright Future Pharmaceutical Laboratories Ltd, Hongkong) for 8 weeks. Demographic, photographic and clinical data for each patient were collected at each visit, and written informed consent was obtained from each patient. This study was approved by the Ethics Committee of XiangYa Hospital, and the protocol is available at the following website: http://www.chictr.org/cn/proj/show.aspx?proj=8045 under the Chinese Clinical Trial Registry registration number: ChiCTR-OCH-14004518. After treatment, patients achieving no less than a PASI50 improvement from baseline ((PASIpost-PASIpre)/PASIpre ≥50%) were defined as responders; otherwise, they were defined as nonresponders^25^. The PASI75 was also used as a cutoff to determine the efficacy of treatment in this study.

### Genotyping

Genomic DNA was extracted from whole blood using the FlexiGene DNA Kit according to the manufacturer’s protocols (Qiagen, Hilden, Germany). In the single nucleotide polymorphism (SNP) discovery phase, 46 DNA samples were genotyped using the Illumina VeraCode ADME Core Panel (Illumina, USA). This VeraCode ADME Core Panel focuses on the standardized PharmADME Core lists and streamlining drug metabolism biomarker analysis. This commercial ADME Core Panel has 184 biomarkers located in 34 genes such as the *ABCB1, SLCO1B1, DPYD*, and *SLC22A1* genes (see Supplementary Table 1). The information in this panel is available from Illumina: http://support.illumina.com.cn/array/array_kits/veracode_adme_core_panel.html. In the validation phase, 105 patients were enrolled, and their positive SNPs were verified using Sequenom Massarray methods (NEW Sequenom MassARRAY 4 System with MassARRAY Nanodispenser). The Hardy-Weinberg equilibrium (HWE) was used to test for population stratification and other forms of nonrandom mating.

### Cell Culture and Plasmid Construction

HEK293 cells were stored in our lab and grown at 37 °C in a humidified 5% CO_2_ atmosphere in Dulbecco’s minimal essential medium (HyClone, USA) supplemented with 10% FBS (BI, Israel), 100 mg/ml penicillin and 100 mg/ml streptomycin (Invitrogen, USA). The pEF6-*SLCO1B1* rs4149056T plasmid was gifted by Dr. Lanxiang Wu. The cloned *SLCO1B1* cDNA rs4149056T and *SLC22A1* rs2282143C genes were inserted into the expression vector pDs-RED N1 according to previously described procedures^26^. Site-directed mutagenesis was performed using PCR to generate the rs4149056C and rs2282143T variants. Details of the primers and enzyme sites for the plasmids are shown in Table 1.

**Table 1:** The primers and enzymes used for genes cloning and RT-PCR.

Gene Name	PRIMER (5′-3′)	Expression Vector pDs-RED N1
*SLCO1B1*-F	GTAAGCTTGGACCAAAATCAACATTTG	HinIII
*SLCO1B1*-R	AACTCGAGACACAATGTGTTTCACTATC	XhoI
*SLC22A1*-F	GTGGTACCATGCCCACCGTGGATGAC	KpnI
*SLC22A1*-R	AACTCGAGTTGGTGCCCGAGGGTTCTGAG	XhoI
*GAPDH F* (RT-PCR)	CTCTGCTCCTCCTGTTCGAC	NA
*GAPDH R* (RT-PCR)	GCCCAATACGACCAAATCC	NA
*SLCO1B1* F (RT-PCR)	CTTCAAATACGTAGAGCAACAGT	NA
*SLCO1B1* R (RT-PCR)	GTAAAAGGACAATGACATCACAG	NA
*SLC22A1* F (RT-PCR)	TCCTGGGAACTGTGCTGG	NA
*SLC22A1* R (RT-PCR)	CAGTTGCCCTTGCTGACC	NA

After the sequence was confirmed, four *SLCO1B1* and *SLC22A1* expression plasmids were successfully constructed and separately transfected into HEK293 cells (HEK293-*SLCO1B1* WT/521 C and HEK293-*SLC22A1* WT/1022 T, respectively) using Turbofect (Thermo Fisher, United States). The pDs-RED N1 vector was also transfected into HEK293 cells (HEK293-MOCK). In this step, 2 μg of each plasmid was transfected into each well of 6 well plates. Then, 36 hours after transfection, acitretin was added into the cells at concentrations from 0 ng/ml to 1000 ng/ml (including 0, 10, 25, 50, 100, 250, 500, and 1000 ng/ml), and the cells were incubated for 5 min, 10 min, or 15 min. Each group was repeated at least 3 times.

Before the acitretin concentration was detected, the harvested cells were dissolved in 250 μl of 10% SDS and maintained at 37 °C for 2 hours. Aliquots (200 μl) were used to determine the intracellular acitretin levels using liquid chromatography-mass spectrometry (LC/MS) methods. The remainder of the lysis was used to determine the protein concentration using BSA as a standard.

### Quantitative realtime PCR analysis

The real-time PCR primers of human *GAPDH*, *SLCO1B1* and *SLC22A1* genes are shown in Table 1; quantitative real-time PCR was performed using a Real Time PCR Detection System, Applied Biosystems 7500 (ABI, USA) and quantified using Taq DNA polymerase (Continental Lab Products, San Diego, CA, USA) according to the manufacturer’s instructions. Reactions were performed for 40 cycles with the following parameters: 94 °C for 45 sec and 60 °C for 45 sec.

### Western blot analysis

The expression of the wild-type and variant *SLCO1B1* and *SLC22A1* proteins was detected with the tag anti-EGFP mAb (Sangon Biotec, Shanghai, China) according to the typical procedure after the plasmids were transfected into the HEK293 cells. A bicinchoninic acid protein assay was used to quantify the total protein in the samples. The quantitative analysis of the integral OD of the bands in the western blot was performed using a Gel-Pro analyzer 4.0 (BIO-RAD, USA).

### HPLC-MS method to detect the acitretin concentration

Standard acitretin was obtained from HuaPont Pharmaceutical Company. Vitamin D_3_ (VD_3_) and acitretin are structurally similar, with a benzene ring structure and long aliphatic hydrocarbon chain. In this study, VD_3_ was taken as the internal standard and was obtained from Sigma (UAS). Methanol, ethyl acetate and methyl tert-butyl ether (MTBE) were obtained from CNW (Germany). As noted in previous publications^27^, a reliable, selective and specific LC/MS method to determine the concentration of acitretin has been developed. Liquid-liquid extraction was used to prepare the cells and plasma samples before detection. In this procedure, 10 μl VD_3_ and 100 μl sample were mixed for 1 min, and then, 1 ml solvent (ethyl acetate-MTBE, 50:50, v:v) was added and mixed. After centrifugation for 5 min, 1 ml supernatant was separated and evaporated to dry under a nitrogen environment. The remaining product was dissolved in 100 μl methanol. The sample acitretin levels were detected with an API 4000 type liquid chromatography-mass spectrometer (API 4000, Sciex AB, Framingham, MA, USA and UFLC-20A, Shimadzu, Kyoto, Japan) using electrospray ionization. In the precursor ion full-scan spectra, the most abundant ions were protonated [M + H]^+^ molecules at m/z 326.9 and 385 for acitretin and Vitamin D_3_, respectively. The product ion spectra of the parent ions showed highly abundant daughter fragments at m/z 177 and 259 for acitretin and Vitamin D_3_. The quantitative limit of detection for acitretin was 1 pg/ml.

### Liquid scintillation counters for transporter assay

To detect the transporter capability of OCT1, the classic substrate [^14^C]-metformin (Moraved Biochemicals and Radiochemicals, CA) was used. Uptake of [3 H]-Estrone-3-sulfate (E3S) by OATP1B1 was detected according to the previous studies. First, 25 × 10^4^ cells were plated in each well at 18–24 hours prior to the accumulation inhibition assay. For the OCT1 transporter assay experiments, KRH buffer (125 mM NaCl, 4.8 mM KCl, 1.2 mM CaCl2, 1.2 mM MgSO4, 1.2 mM KH2PO4, 25 mM HEPES, 5.6 mM glucose, pH 7.4) was used. The cells were washed once with prewarmed KRH buffer at 37 °C for 15 min. Metformin was taken as a positive transporter substrate. After incubation with KRH buffer containing different concentrations of acitretin or 10 μM [^14^C]-metformin and 40 μM unlabeled metformin for 10 min, the uptake was halted by removing the KRH buffer and washing the cells with ice-cold KRH buffer 3 times. The cells were solubilized in 0.1 N sodium hydroxide (NaOH) and shaken for 30 min with 0.1 N hydrochloric acid (HCL) to neutralize the buffer. Then, 300 μl cell lysate was transferred to a scintillation tube containing 3 ml Biodegradable Counting Cocktail buffer (Fisher Scientific Inc., Pittsburgh, PA). The radioactivity was counted using a multipurpose scintillation counter (Beckman LS6500 Counter, Brea, CA). The protein concentrations were measured using a BCA protein assay kit (Bio-Rad Co. Hercules, CA), which was also employed to normalize the radioactivity values using a method based on previous literature^26,28^.

### Data statistics and analysis

The analyses were performed using the SPSS 18.0 statistical package (IBM SPSS, Illinois). The frequency distribution of genotypes and alleles in different subgroups were tested by ANOVA and chi square analysis. The acitretin levels in different groups were analyzed by a dependent T-test. A two-tailed *P* value less than 0.05 was considered significant. The significance was adjusted with the Bonferroni correction in multiple tests. The post hoc power of the sample size in the chi square analysis was calculated with G. power (versions 3.1.9.2): the effect sizes were 1.024 and 0.417 and the sample sizes were 46 and 105 for the discovery and validation phases, respectively, and the α error was 0.05 and the df was 5; thus, the power was over 0.999 to 1.000.

All methods were performed in accordance with the relevant guidelines and regulations. All data generated or analyzed during this study were included in this article (and Supplementary Information).

## Results

### The epidemiological data and clinical efficacy

There was no significant difference in the average PASI baseline values (11.28 ± 4.85 vs. 11.01 ± 4.79, *P* = 0.832), BMI (23.15 ± 3.09 vs. 22.73 ± 3.34, *P* = 0.574) or age (40.96 ± 15.54 vs. 38.75 ± 15.25, *P* = 0.410) of the subjects between the discovery and validation phases. In both phases, the BMI and PASI baseline values were not significantly different between the responder and nonresponder groups. However, the age showed a significant difference (35.30 ± 15.67 vs. 42.45 ± 14.0 years, *P* = 0.013) between the responders and nonresponders. The average age of patients with different genotypes was not notably different (Supplementary Table 2). According to the PASI50 cutoff, 23 (50.0%) patients were responders and 23 (50.0%) were nonresponders in the discovery phase; there were 48 (45.7%) responders and 57 (54.3%) nonresponders in the validation phase. Combining the data for both phases, 71 (47.02%) participants showed a PASI50 improvement after 8 weeks of treatment. Only 41 (27.15%) patients achieved PASI75 improvement, and 110 (72.85%) patients were classified as responding poorly when using the PASI75 cutoff.

### The association between the polymorphisms and efficacy

In the discovery phase, 12 patients had the *SLCO1B1* rs4149056TC (c.521TC) genotype and 34 patients had the rs4149056TT genotype. Allele T of rs4149056 was associated with an ineffective response (*P* = 0.013, OR = 6.611). The difference was significant with a *P* = 0.05 threshold, although this difference was no longer significant after Bonferroni correction (*P* > 0.00027). The same tendency was confirmed during the validation phase: the T allele was more frequent in the ineffective group than in the effective group (95.65% vs. 78.26%, *P* = 0.028). For the *SLC22A1* gene, the rs2282143C (c.1022 C) allele was more frequent in the ineffective group than in the effective group in the discovery phase (93.19% vs. 67.39%, *P* = 0.002) and validation phase (90.35% vs. 78.13%, *P* = 0.014), when the patients received the same treatment. Combining the data from the discovery and validation phases, there was still a strong correlation between the rs4149056 and rs2282143 polymorphisms and the efficacy of acitretin (*P* = 0.001, and *P* < 0.001, respectively). The details are shown in Table 2. No other genetic variations of metabolic enzymes, such as CYP3A, CYP2C, CYP29C, or CYP26, were found to be associated with the efficacy of acitretin in our study.

**Table 2:** Relationship between the rs4149056T > C and rs2282143C > T polymorphisms and acitretin efficacy in patients, based on PASI50.

Phase	*SLC22A1* Rs2282143C > T						*SLCO1B1* Rs4149056T > C					
	Genotype/allele	PASI < 50	PASI > 50	P value	OR[95CI]	HWE P value	Genotype/allele	PASI < 50	PASI > 50	P value		HWE P value
Discovery Phase	CC	19	8	*P* < 0.001	OR:11.875 [2.677,52.670]	*P* = 0.368	TT	21	13	*P* = 0.007	OR:8.077 [1.523, 42.834]	*P* = 0.309
	CT + TT	3	14 + 1				TC	2	10			
	C	41	30	*P* = 0.002	OR:6.613 [1.759, 24.866]		T	44	36	*P* = 0.013	OR:6.111 [1.258, 29.693]	
	T	3	16				C	2	10			
Validation Phase	CC	47	27	*P* = 0.003	OR:3.656 [1.502, 8.897]	*P* = 0.859	TT	52	37	*P* = 0.003	OR:8.027 [1.681, 38.338]	*P* = 0.428
	CT + TT	9 + 1	19 + 2				TC	4	11			
	C	103	73	*P* = 0.014	OR:2.622 [1.192, 5.764]		T	108	85	*P* = 0.028	OR:3.44 [1.075, 11.361]	
	T	11	23				C	4	11			
Total samples	CC	66	35	*P* < 0.001	OR:5.222 [2.454, 11.111]	*P* = 0.702	TT	73	50	*P* < 0.001	OR:5.110 [1.925, 13.561]	*P* = 0.223
	CT + TT	12 + 1	33 + 3				TC + TT	6	21			
	C	144	103	*P* < 0.001	OR:3.895 [2.011, 7.542]		T	152	121	*P* = 0.001	OR:4.397 [1.721, 11.235]	
	T	14	39				C	6	21			

Notes: In the discovery phase, 45/46 patients were genotyped with rs2282143C > T; in the validation phase, 104/105 patients were genotyped with rs4149056T > C.

When PASI75 was used as the responsiveness cutoff, there were 13 (28.26%) responders and 33 (71.74%) nonresponders in the discovery phase, and 28 (26.67%) responders and 77 (73.33%) nonresponders in the validation phase. The same association was found between the rs4149056 and rs2282143 polymorphisms and the efficacy of acitretin (*P* < 0.001 and *P* = 0.001, respectively) (Table 3).

**Table 3:** Relationship between the rs4149056T > C and rs2282143C > T polymorphisms and acitretin efficacy in patients, based on PASI75.

Phase	*SLC22A1* rs2282143C > T						*SLCO1B1* rs4149056T > C					
	Genotype/allele	PASI < 75	PASI > 75	P value	OR[95CI]	HWE P value	Genotype/ allele	PASI < 75	PASI > 75	P value	OR[95CI]	HWE P value
Discovery Phase	CC	22	5	*P* = 0.130	2.800[0.721, 10.874]	*P* = 0.368	TT	28	6	*P* = 0.007	6.533[1.537, 27.776]	*P* = 0.309
	CT + TT	11	6 + 1				TC	5	7			
	C	55	18	*P* = 0.132	2.222[0.774, 6.381]		T	61	19	*P* = 0.013	4.495[1.278, 15.812]	
	T	11	8				C	5	7			
Validation Phase	CC	60	14	*P* = 0.006	3.529[1.412, 8.820]	*P* = 0.859	TT	70	19	*P* = 0.002	5.526[1.749, 17.466]	*P* = 0.428
	CT + TT	16 + 1	12 + 2				TC	6	9			
	C	136	40	*P* = 0.003	3.022[1.413, 6.463]		T	146	47	*P* = 0.003	4.660[1.576, 13.776]	
	T	18	16				C	6	9			
Total samples	CC	82	19	*P* = 0.002	3.237[1.522, 6.883]	*P* = 0.702	TT	98	25	*P* < 0.001	5.702[2.355, 13.807]	*P* = 0.209
	CT + TT	27 + 1	18 + 3				TC	11	16			
	C	191	56	*P* = 0.001	2.823[1.522, 5.234]		T	207	66	*P* < 0.001	4.562[2.017, 10.318]	
	T	29	24				C	11	16			

Notes: In the discovery phase, 45/46 patients were genotyped with rs2282143C > T; in the validation phase, 104/105 patients were genotyped with rs4149056T > C. The OR value was used to establish significance based on the 95% CI. If the 95% CI contains the value 1, equivalent to P > 0.05, there is no significance; otherwise, the data are significant. A 95% CI upper limit < 1 indicates a protective factor; a lower limit > 1 indicates a risk factor. For example, in the validation phase, the probability of inefficacy/efficacy of the C allele was 3.473 times and the lower limit of the 95% CI was 1.619 relative to that of the mutant T allele, indicating that the C site was a risk factor for inefficacy.

### The expression of the SLCO1B1/SLC22A1 genes containing the wild-type or variant alleles

**Figure 1:**
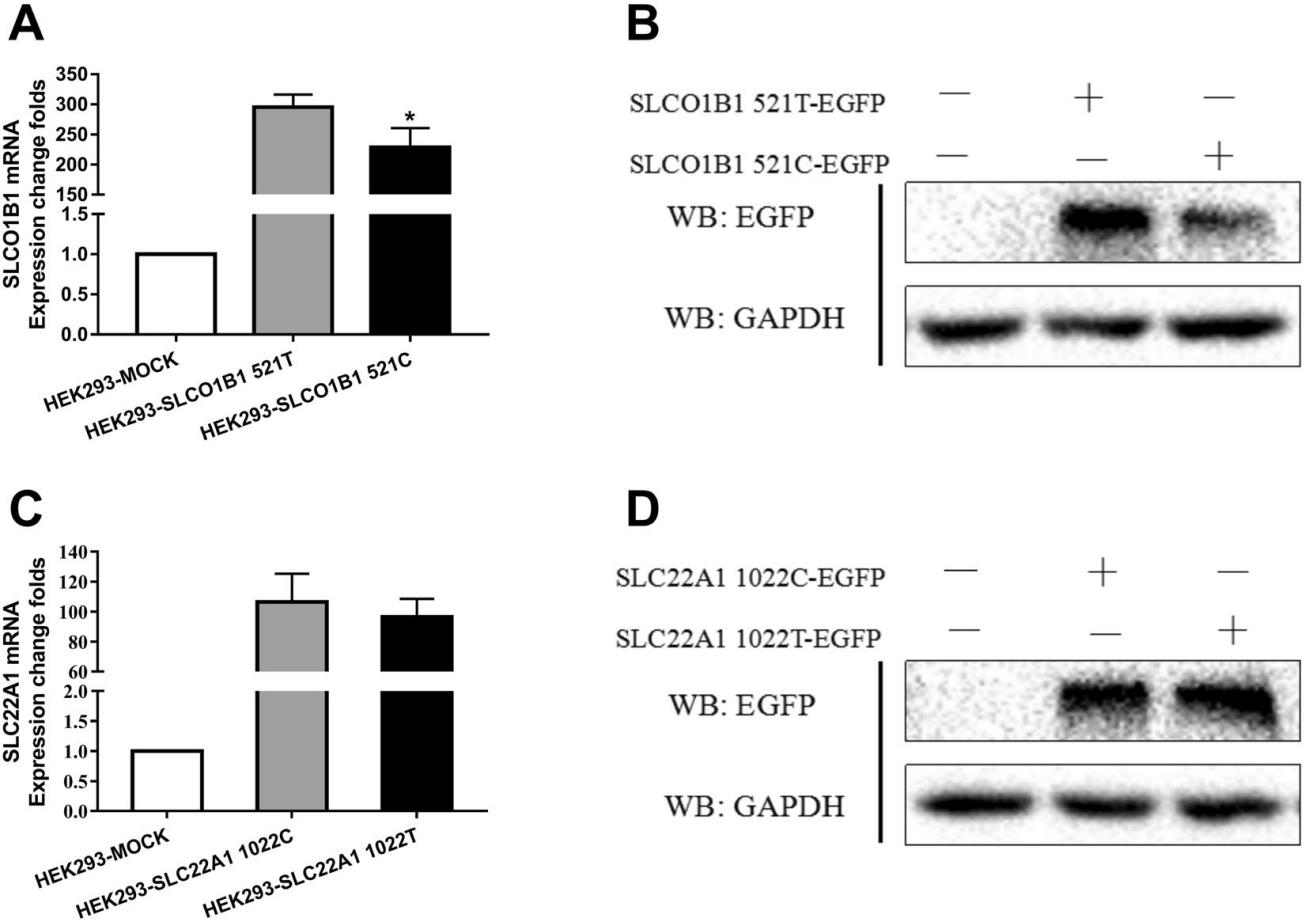
Expression of *SLCO1B1* and *SLC22A1* in HEK293 cells with or without transfection. Legend: (**A**,**B**) mRNA and protein of *SLCO1B1* expressed in HEK293 cells transfected with different alleles of *SLCO1B1* rs4149056T/C; (**B**,**C**,**D**) mRNA and protein of *SLC22A1* expressed in HEK293 cells transfected with different alleles of *SLC22A1* rs2282143C/T. GAPDH was used as the internal control.

RT-PCR and western blotting were used to detect the mRNA and protein expression of both genes containing the different alleles. The rs4149056C allele significantly reduced the transcription level of the *SLCO1B1* gene (rs4149056T and C, 294.72 ± 10.69 vs. 227.36 ± 19.94, *P* = 0.012), as well as the protein expression. There was no significant difference in *SLC22A1* mRNA expression between the rs2282143T and C alleles (rs2282143T and C, 96.38 ± 12.09 vs. 106.46 ± 18.84, *P* = 0.4365) or in the protein expression (Fig. 1).

### The transporter ability of OATP1B1 and OCT1 transporters

[^3^H]-Estrone-3-sulfate (E_3_S) is a classic substrate of OATP1B1, and rifampicin acts as an OATP1B1 inhibitor that can be used to detect the transporter function of OATP1B1. As noted above, the plasma acitretin concentration ranges from 87 to 1358 ng/ml in psoriasis patients. In this study, acitretin did not inhibit HEK293 cell proliferation or viability even at 10000 ng/ml (Supplementary Figure S2). In accordance with the preliminary experiments, we chose incubation concentrations from 5 to 1000 ng/ml for the acitretin uptake experiments. When the concentration was less than 100 ng/ml, the uptake ability was almost saturated at 10 min, whereas at a higher concentration (>100 ng/ml), the saturation time was 15 min (Supplementary Figure S3). In this study, an incubation time of 15 min was taken as the endpoint.

**Figure 2:**
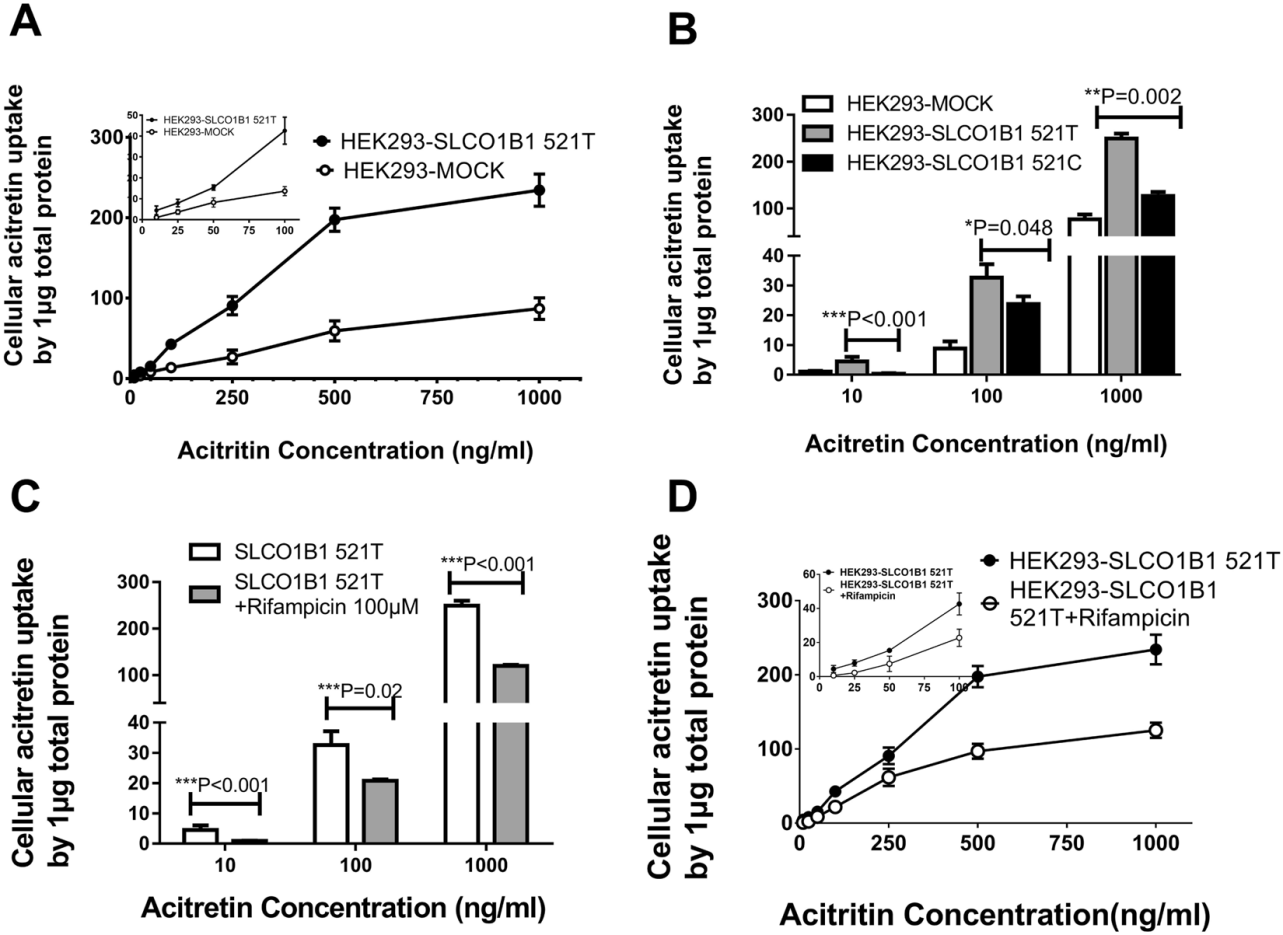
Cellular levels of acitretin with different *SLCO1B1* rs4149056 alleles. Legend: (**A**) Uptake of acitretin was significantly higher in HEK293-*SLCO1B1* cells than in HEK293-MOCK cells. (**B**) Uptake mediated by *SLCO1B1* 521 C allele was less than that in the wild-type 521 T cells. (**C**,**D**) Uptake of acitretin mediated by SLCO1B1 was inhibited by rifampicin. *SLCO1B1* 521 T is the wild-type allele, and 521 C is the variant.

In this study, the [^3^H]-E_3_S concentration was markedly higher (~5.5-fold) in the HEK293-*SLCO1B1* cell group than in the HEK293-MOCK cell group. With *SLCO1B1* overexpressed, acitretin (5–1000 ng/ml) uptake in HEK293-*SLCO1B1* cells was significantly higher than the uptake in HEK293-MOCK cells (*P* < 0.001). Acitretin uptake by HEK293-*SLCO1B1*C 521 C cell was significantly lower than that by wild-type *SLCO1B1* 521 T cell (*P* < 0.05). Meanwhile, the uptake of acitretin was significantly inhibited by rifampicin (*P* < 0.05; Fig. 2).

**Figure 3:**
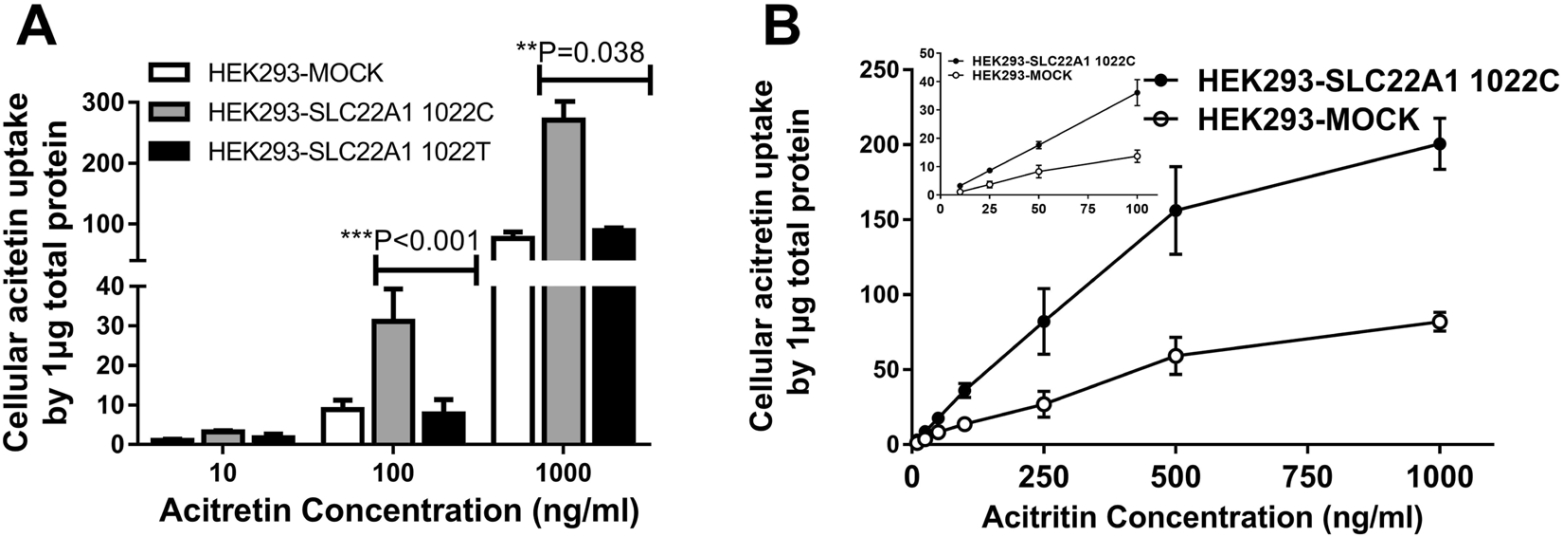
Uptake of [^14^C]-metformin in HEK293-*SLC22A1* cells expressing different alleles. Legend: Uptake of [^32^S]-metformin by the *SLC22A1* 1022 C/T alleles. With 5 or 10 μM [^14^C]-metformin incubation, there was no significant difference in uptake between the two alleles. As the concentration increased, the uptake by *SLC22A1* 1022 T was less than that by the wild-type 1022 C allele. *SLC22A1* 1022 C is the wild-type allele, and 1022 T is the variant for this polymorphism.

**Figure 4:**
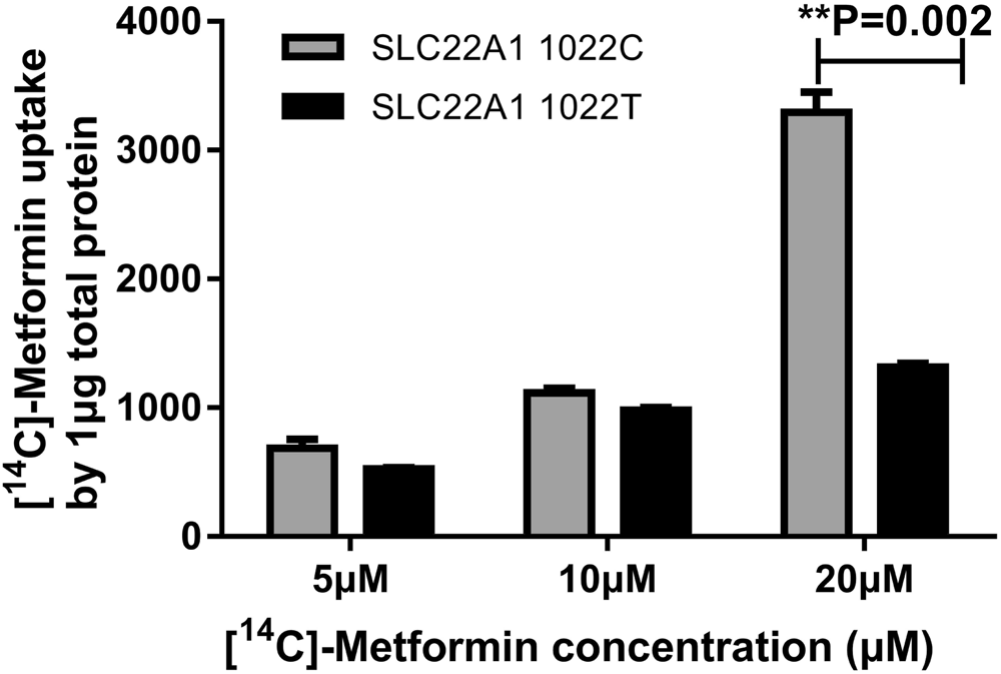
Cellular levels of acitretin with different *SLC22A1* rs2282143 alleles. Legend: (**A**) Uptake of acitretin was significantly higher in HEK293-*SLC22A1* cells than in HEK293-MOCK cells. (**B**) Uptake of acitretin mediated by *SLC22A1* 1022 T was less than that by wild-type 1022 C.

After overexpression of the *SLC22A1* gene, the concentration of the classic cellular substrate metformin increased remarkably compared to that in the HEK293-MOCK cells. Compared with the wild-type allele, the rs2282143T allele significantly reduced the uptake of [^32^S]-metformin, especially at a concentration of 20 μM (3073.29 ± 409.11 vs. 1316.21 ± 49.14, *P* = 0.002; Fig. 3). As with the classic substrate metformin, HEK293-*SLC22A1* cells showed significantly higher uptake of acitretin than HEK-MOCK cells. The variant rs2282143T allele significantly decreased the transport ability of acitretin compared with the wild-type rs2282143C allele (Fig. 4). We also analyzed the transport ability of acitretin at an incubation time of 10 min, and a similar acitretin uptake trend was observed in the different variant groups.

## Discussion

The efficacy of acitretin varies markedly among different individuals with psoriasis, possibly due to the increased bioavailability^29^. Young *et al*. reported that *VEGF* gene polymorphisms were associated with the response to acitretin in psoriasis patients^30^. In this study, we discovered that the rs4149056 and rs2282143 polymorphisms affected the treatment outcomes. *In vitro*, we confirmed that acitretin was transported into the cells by *SLCO1B1* and *SLC22A1* transporters, and the rs4149056C and rs2282143T alleles reduced the respective drug transporter capability.

Oral acitretin and topical calcipotriol are used as a common combination regimen to treat moderate to severe psoriasis to increase the efficacy of acitretin and decrease adverse reactions. Calcipotriol is administered topically and does not affect the pharmacokinetics of acitretin^18,31^. Acitretin combines with the nuclear receptors RAR and RXR, and the nuclear receptor is more conserved. We previously investigated the relationship between RAR/RXR polymorphisms and the efficacy of acitretin, but found no positive results (not yet published). Therefore, in this experiment, we focused on acitretin pharmacokinetics, especially the impacts of polymorphisms in the *SLCO1B1* and *SLC22A1* genes.

An SNP that results in an amino-acid change is more likely to impact protein functions. *SLCO1B1* rs4149056T/C encodes for an amino acid substitution from valine to arginine at residue 147 (p.V147L). This variant significantly decreases the expression of the *SLCO1B1* gene and transport of classic substrates such as statins, bilirubin, E_3_S and repaglinide^12,32,33^. Reduced transporter ability may lead to an elevated circulating concentration of the drug, as less substrate is transported into the hepatocytes for clearance and more drug is maintained in the plasma. Our data demonstrated that the rs4149056C allele markedly decreased the uptake of acitretin. Therefore, patients who carry the rs4149056C allele showed good drug responsiveness, which is consistent with the previously published conclusion^34^. For patients carrying the rs4149056T allele, achieving the same efficacy requires increasing the acitretin dosage or selecting other therapies.

The protein of *SLC22A1* gene–OCT1 is expressed in the basolateral membrane of epithelial cells in the liver and intestine. In *SLC22A1*(−/−) mice, the hepatic uptake, intestinal excretion and renal secretion of the substrates have been reported to be greatly reduced, and increased drug sensitivity have been found^35^. In the *SLC22A1* gene, several polymorphisms have been identified that alter the clinical responses to metformin or imatinib and the progression of several diseases^21,36,37^. The polymorphic variant rs2282143T has been shown to decrease the uptake of lamivudine and [^14^C]-TEA by 48.7% and 35.9%, respectively^22,23^. In this study, we confirmed that the rs2282143T allele decreased the uptake of substrates, including [^14^C]-metformin and acitretin. Patients who carry rs2282143T alleles were shown to be better responders when treated with acitretin. The T allele of rs2282143 did not significantly decrease the mRNA or protein expression of the *SLC22A1* gene but did reduce the uptake of substrates. The possible mechanism is unclear. In rs2282143, the C allele leads to an amino acid change from proline to leucine. The three-dimensional structure of this protein may be changed, potentially into a drug-linked pouch structure, which may affect drug binding and transport. Determining the possible mechanism requires further research.

We established that acitretin was a substrate of the transporters OATP1B1 and OCT1. OATP1B1 transports a variety of substrates that contain organic anions, including thyroid hormones T3 and T4, prostaglandin E2, MTX, and statins. Similar to other acidic drugs, acitretin has one carboxylic acid group and easily dissociates into an anionic structure *in vivo*. Acitretin has also been shown to be transported by OCT1 *in vitro*, but the intake mechanism is not clear. Few studies have focused on acitretin, and whether acitretin forms a cationic compound in the body remains unclear. In this study, the *SLCO1B1* rs4149056 and *SLC22A1* rs2282143 polymorphisms were shown to affect the uptake and clinical efficacy of acitretin. These findings may partly explain the drug response variation. Our pharmacogenetics study provided some important suggestions for precision medicine in psoriasis. Patients who carry the rs4149056T or rs2282143C alleles may require a higher dosage of acitretin or selection of another therapeutic regimen to achieve the same efficacy as patients with variant alleles.

We found a weak relationship between the rs2282143C > T and rs4149056T > C SNPs in the total sample (r^2^ = 0.369). There were correlations between rs2282143 and another gene polymorphism, rs12248560 (r^2^ = 0.370). This relationship may suggest that there was some overlap in the polymorphisms. In this study, we reanalyzed the rs2282143 and rs4149056 haplotypes and found that the patients with the rs2282143CT-rs4149056TC haplotype were prone to be more responsive. Details are provided in Supplementary Table 4.

This study has some limitations. First, the plasma and subcutaneous acitretin concentrations were not detected during treatment. Acitretin is unstable and prone to decomposition if exposed to light for more than 10 min. Second, the clinical observation of this trial lasted for only 8 weeks. We inferred that the effectiveness would be improved with longer treatment. However, it was difficult for us to obtain therapeutic information for more than 12 weeks of treatment in patients with poor efficacy. Most of our patients had a long course of disease and treatment. Once the efficacy appears poor, patients may change their therapy on their own and withdraw from the trial. Third, only 41 and 22 patients of the patients achieved PASI75 and PASI90 improvements, respectively, and the sample size was too small to obtain sufficient power for genotyping and analysis. Meanwhile, 186 genetic biomarkers were screened in 46 samples. Thus, there may be some positive SNPs omitted due to the limited number of samples.A larger study sample is needed to verify the roles of *SLCO1B1* and *SLC22A1* polymorphisms in the variability in drug disposition, responsiveness and toxicity. In addition, calcipotriol ointment was combined with acitretin to treat psoriasis in this study. Calcipotriol improved the disease on its own but the amount of calcipotriol used topically was difficult to quantify. Therefore, the use of calcipotriol may have affected the analysis to some degree.

## Conclusion

The *SLCO1B1* rs4149056 and *SLC22A1* rs2282143 polymorphisms affect the clinical efficacy of acitretin in the treatment of psoriasis. The functional experiments indicates that acitretin can be pumped into cells by the SLCO1B1 and SLC22A1 transporters, and that the rs4149056C and rs2282143T alleles reduce the uptake of acitretin, and are significantly associated with clinical effective responsiveness in psoriasis patients when treated by acitretin.

## Electronic supplementary material

Supplementary

## Data Availability

The data included in this manuscript is available.
