# Supplementary material for: Polymorphisms of SLCO1B1 rs4149056 and SLC22A1 rs2282143 are associated with responsiveness to acitretin in psoriasis patients
Source: Sci Rep. 2018 Sep 4;8:13182. doi: 10.1038/s41598-018-31352-2 (PMC6123456; doi:10.1038/s41598-018-31352-2)
Supplement: Supplementary file 1 — Supplementary [file 41598_2018_31352_MOESM1_ESM.pdf]

Polymorphisms of *SLCO1B1* rs4149056 and *SLC22A1* rs2282143 are associated with responsiveness to acitretin in psoriasis patients

Wangqing Chen1,2,3, Xu Zhang1,3, Wei Zhang2, Cong Peng1,3, Wu Zhu*1,3, Xiang Chen*1,2,3

1. Department of Clinical Pharmacology, Xiangya Hospital, Central South University, ChangSha, Hunan, 410008, China

2. Department of Dermatology, Xiangya Hospital, Central South University, Ch angsha, Hunan, 410008, China

3. Hunan Key Laboratory of Skin Cancer ans Psoriasis, ChangSha, Hunan, 410 008, China

Corresponding author: Xiang Chen and Wu Zhu

Address: Xiangya Road No.87, Xiangya Hospital, Changsha City, Hunan Provin ce, 410008, China; E-mail: chenxiangck@126.com; or zhuwu70@hotmail.com

The word counts for the abstract (188), text (3970), number of references (33), tables (3), Figures (4), Supplementary Tables (4), Supplementary Figures (3).

Funding:

This project was supported by The Special Foundation for State Major Basic Research Program of China (No. 81430075) and the National Natural Science Foundation of China (No. 81573049 and 81773329).

Conflicts of interest

All the authors confirmed that there were no conflicts of interest.

Patient 1

Patient 2

Patient 3

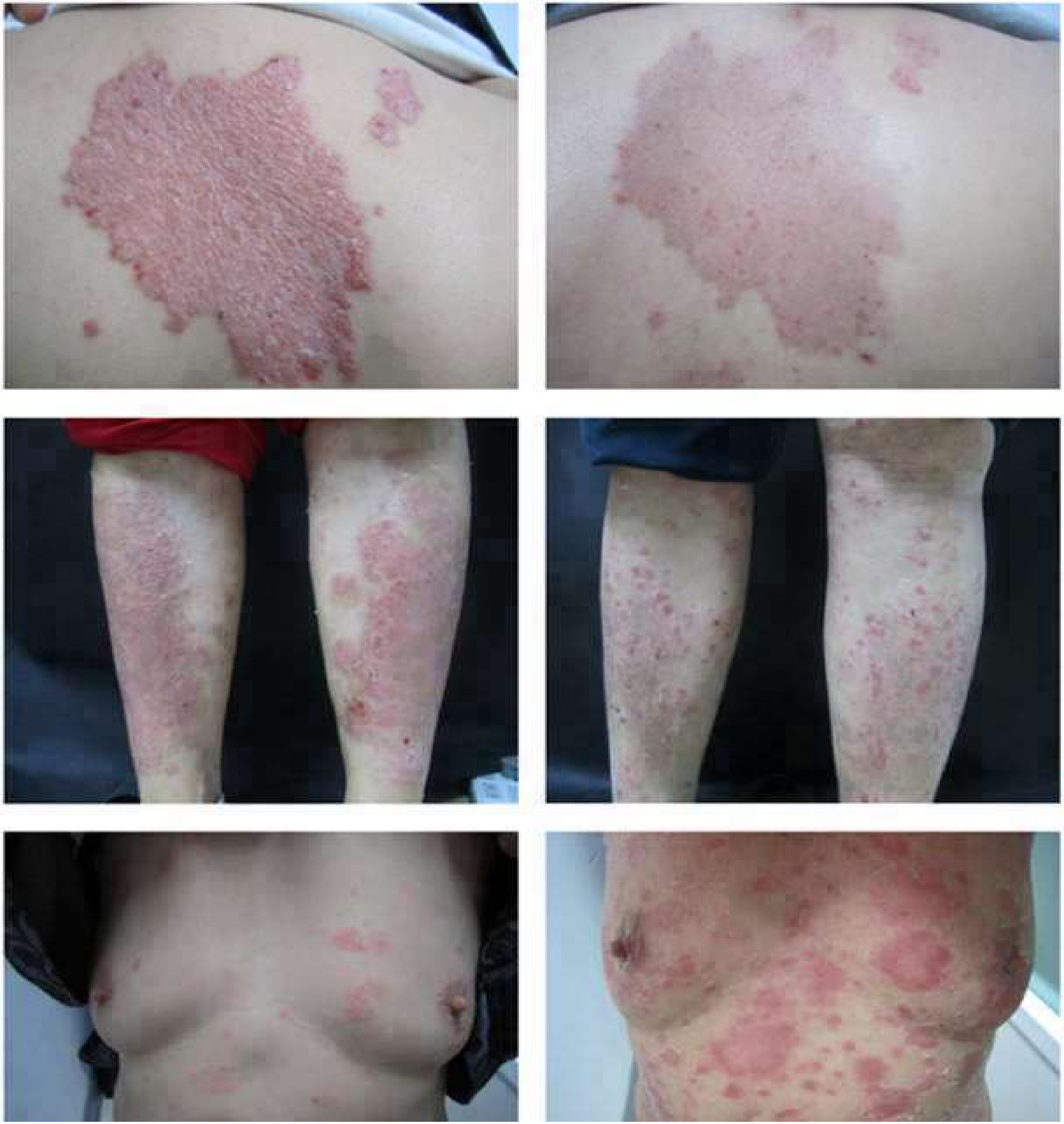

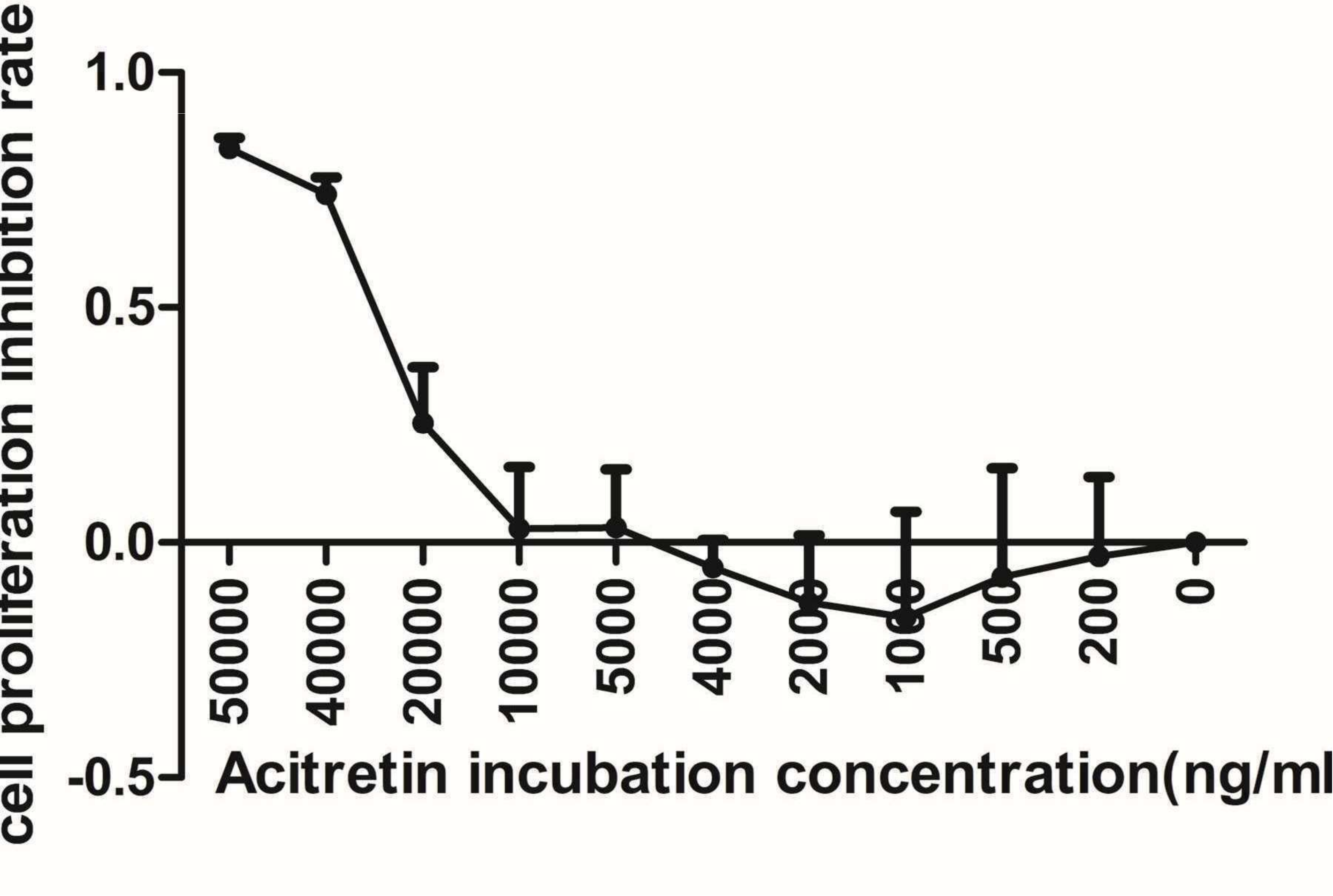

cell proliferation inhibition rate
1.0
0.5
0.0
-0.5
500000
400000
200000
100000
50000
40000
2000
1000
500
200
0
Acitretin incubation concentration(ng/ml

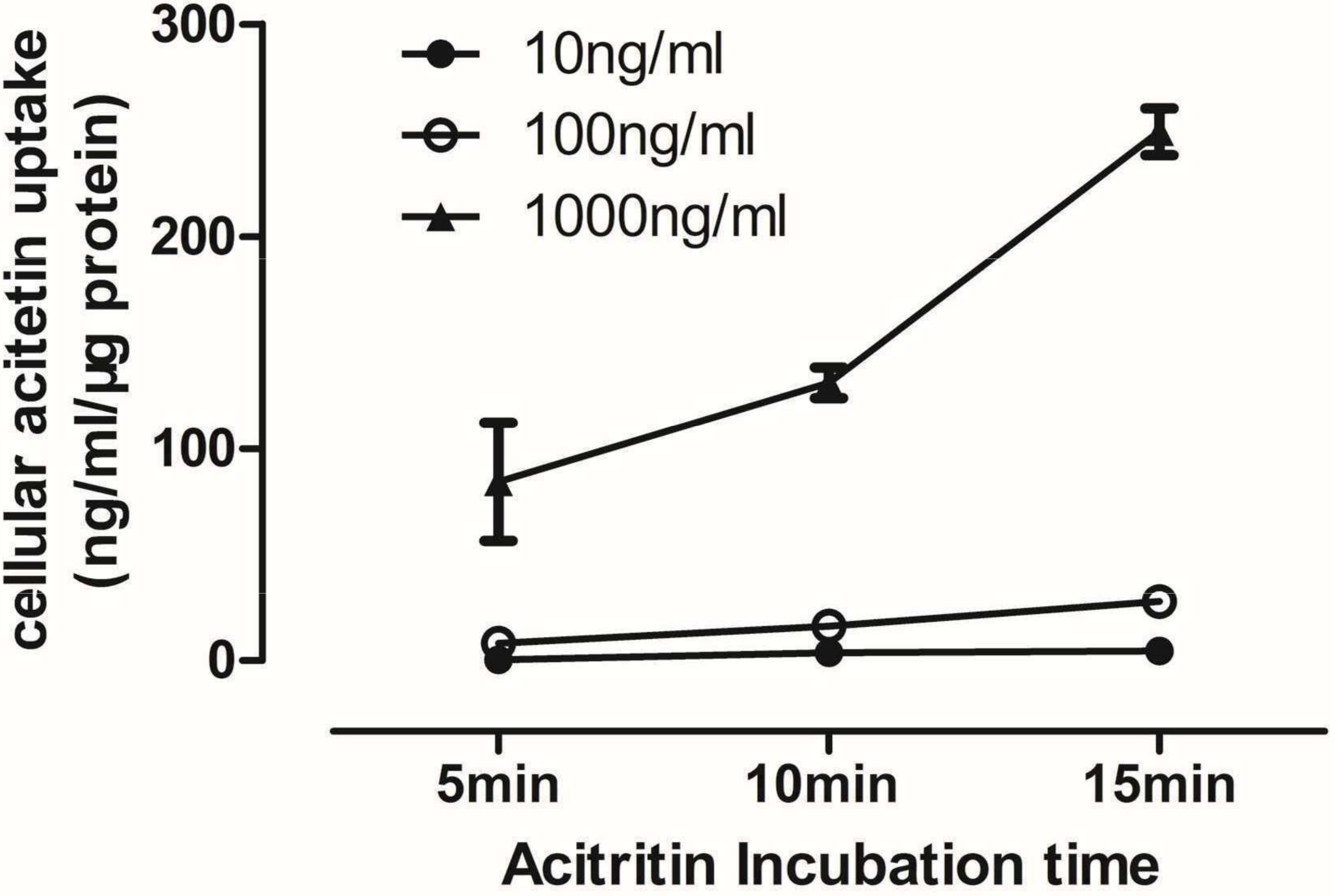

300
200
100
0
cellular acitetin uptake
(ng/ml/µg protein)
10ng/ml
100ng/ml
1000ng/ml
5min
10min
15min
Acitritin Incubation time

Legends of Supplementary Figures

Supplementary Figure S1. Efficacy of acitretin varied in psoriasis patients

Legends: All patients were treated with acitretin 30 mg/day plus calcipotriol ointment. With treatment for 8 weeks, patient 1 was classed as a responder with PASI improvement >50%, and patient 2 was classed as a non-responder with PASI improvement <50%. Patient 3 was classed as a non-responder, with markedly increased disease severity after 1-week treatment with acitretin.

Supplementary Figure S2. The effect of different acitretin concentration incubation on HEK293 cells proliferation.

Legends: With acitretin concentration no more than 200 ng/ml, there was no significant influence on cell proliferation. While, when the concentration was up to 500 ng/ml, there were notable changes in cell proliferation.

Supplementary Figure S3. Incubation time 15min for acitretin uptake

Legends: As the incubation time increased, cellular acitretin uptake was saturated at 10 min with a concentration of 10 or 100 ng/ml. When acitretin concentration reached 1000 ng/ml, the uptake capability was still increasing at 15 min.

Supplement Table 1. The SNPs list of vera code adme core panel					
**Assay**	**Gene Symbol**	**PrimaryName**	**Nucleotide Change**	**Effect**	**RefSeq ID**
1	ABCB1	1236C>T	c.1236C>T	-	rs1128503
2	ABCB1	-129 T>C)	c.-129C>T	-	rs3213619
3	ABCB1	2677nt G>T>A	c.2677G>T>A	-	rs2032582
4	ABCB1	C3435ntT	c.3435C>T	-	rs1045642
5	ABCC2	-24C>T	c.-24C>T	-	rs717620
6	ABCC2	A1450T	c.4348G>A	p.A1450T	rs56296335
7	ABCC2	I1324I	c.3972C>T	p.I1324I	rs3740066
8	ABCC2	R768W	c.2302C>T	p.R768W	rs56199535
9	ABCC2	S789F	c.2366C>T	p.S789F	rs56220353
10	ABCC2	V417I	c.1249G>A	p.V417I	rs2273697
11	ABCG2	421nt C>A	c.421C>A	p.Q141K	rs2231142
12	ABCG2	Q126X	c.376C>T	p.Q126X	rs72552713
13	CYP1A1	*2 (I462V)	2455A>G	p.I462V	rs1048943
14	CYP1A1	*3	3205T>C	-	rs1800031
15	CYP1A1	*4	2453C>A	p.T461N	rs1799814
16	CYP1A1	*5	2461C>A	p.R464S	rs41279188
17	CYP1A1	*6	1636G>T	p.M331I	rs56313657
18	CYP1A1	*7	2346-2347insT	Frameshift	rs72547510
19	CYP1A1	*8	2414T>A	p.I448N	rs72547509
20	CYP1A2	*1C	-3860G>A	-	rs2069514
21	CYP1A2	*1F	-163C>A	-	rs762551
22	CYP1A2	*1K	-729C>T	-	rs12720461
23	CYP1A2	*7	3533G>A	Splicing defect	rs56107638
24	CYP2A6	*11	c.670T>C	p.S224P	rs28399447
25	CYP2A6	*17	c.1093G>A	p.V365M	rs28399454
26	CYP2A6	*1B	-	-	-
27	CYP2A6	*2	c.479T>A	p.L160H	rs1801272
28	CYP2A6	*20	c.587-588delAA	Frameshift	rs28399444
29	CYP2A6	*4			NA
30	CYP2A6	*6	c.383G>A	p.R128Q	rs4986891
31	CYP2A6	*7	c.1412T>C	p.I471T	rs5031016
32	CYP2A6	*8	c.1454G>T	p.R485L	rs28399468
33	CYP2A6	*9	g.-48T>G	TATA box	rs28399433
34	CYP2B6	*16	c.983T>C	p.I328T	rs28399499
35	CYP2B6	*2	c.64C>T	p.R22C	rs8192709
36	CYP2B6	*28	c.1132C>T	p.R378X	rs34097093
37	CYP2B6	*8	c.415A>G	p.K139E	rs12721655
38	CYP2B6	*9 (*6)	c.516G>T	p.Q172H	rs3745274
39	CYP2C19	*12	c.1473A>C	p.X491C; 26 extra AA	rs55640102
40	CYP2C19	*17	g.-806C>T	-	rs12248560

41	CYP2C19	*2	c.681G>A	Splicing defect	rs4244285
42	CYP2C19	*3	c.636G>A	p.W212X	rs4986893
43	CYP2C19	*4	c.1A>G	GTG start codon	rs28399504
44	CYP2C19	*5	c.1297C>T	p.R433W	rs56337013
45	CYP2C19	*6	c.395G>A	p.R132Q	rs72552267
46	CYP2C19	*7	g.19294T>A	Splicing defect	rs72558186
47	CYP2C19	*8	c.358T>C	p.W120R	rs41291556
48	CYP2C8	*2	c.805A>T	p.I269F	rs11572103
49	CYP2C8	*3	c.1196A>G	p.K399R	rs10509681
50	CYP2C8	*4	c.792C>G	p.I264M	rs1058930
51	CYP2C8	*5	c.475delA	Frameshift	rs72558197
52	CYP2C8	*7	c.556C>T	p.R186X	rs72558195
53	CYP2C8	*8	c.556C>G	p.R186G	rs72558195
54	CYP2C9	*10	c.815A>G	p.E272G	rs9332130
55	CYP2C9	*11	c.1003C>T	p.R335W	rs28371685
56	CYP2C9	*12	c.1465C>T	p.P489S	rs9332239
57	CYP2C9	*13	c.269T>C	p.L90P	rs74052158
58	CYP2C9	*15	c.485C>A	p.S162X	rs72558190
59	CYP2C9	*2	c.430C>T	p.R144C	rs1799853
60	CYP2C9	*25	c.353-362delAGAAATGGAA	Frameshift	rs72558188
61	CYP2C9	*3	c.1075A>C	p.I359L	rs1057910
62	CYP2C9	*4	c.1076T>C	p.I359T	rs56165452
63	CYP2C9	*5	c.1080C>G	p.D360E	rs28371686
64	CYP2C9	*6	818delA (cDNA)	Frameshift	rs9332131
65	CYP2C9	*8	c.449G>A	p.R150H	rs7900194
66	CYP2C9	*9	c.752A>G	p.H251R	rs2256871
67	CYP2D6	*10	100C>T	p.P34S	rs1065852
68	CYP2D6	*11	883G>C	Splicing defect	rs5030863
69	CYP2D6	*12	124G>A	p.G42R	rs5030862
70	CYP2D6	*14	1758G>A	p.G169R	rs5030865
71	CYP2D6	*15	137-138insT	Frameshift	rs72549357
72	CYP2D6	*17	1023C>T	p.T107I	rs28371706
73	CYP2D6	*18	4125-4133insGTGCCCACT	p.468-470dupVPT	NA
74	CYP2D6	*19	2539-2542delAACT	Frameshift	rs72549353
75	CYP2D6	*20	1973-1974insG	Frameshift	rs72549354
76	CYP2D6	*21	2573-2574insC	Frameshift	rs72549352
77	CYP2D6	*2a	-1584C>G		rs1080985
78	CYP2D6	*3	2549delA	Frameshift	rs35742686
79	CYP2D6	*38	2587-2590delGACT	Frameshift	rs72549351
80	CYP2D6	*4	1846G>A	Splicing defect	rs3892097

81	CYP2D6	*41	2988G>A	Splicing defect	rs28371725
82	CYP2D6	*42	3259-3260insGT	Frameshift	rs72549346
83	CYP2D6	*44	2950G>C	Splicing defect	rs72549349
84	CYP2D6	*5			NA
85	CYP2D6	*56	3201C>T	p.R344X	rs72549347
86	CYP2D6	*6	1707delT	Frameshift	rs5030655
87	CYP2D6	*7	2935A>C	p.H324P	rs5030867
88	CYP2D6	*8	1758G>T	p.G169X	rs5030865
89	CYP2D6	*9	2615-2617delAAG	p.K281del	rs5030656
90	CYP2E1	*2	1132G>A	p.R76H	rs72559710
91	CYP3A4	*1G	IVS10+12G>A	-	rs2242480
92	CYP3A4	*2	c.664T>C	p.S222P	rs55785340
93	CYP3A4	*20	c.1461-1462insA	Frameshift	rs67666821
94	CYP3A4	*6	c.830-831insA	Frameshift	rs4646438
95	CYP3A5	*10(F446S)	29753T>C	c.F446S	rs41279854
96	CYP3A5	*3	6986A>G	Splicing defect	rs776746
97	CYP3A5	*5	12952T>C	Splicing defect	rs55965422
98	CYP3A5	*6	14690G>A	Splicing defect	rs10264272
99	CYP3A5	*7	27131-27132insT	Frameshift	rs41303343
100	DPYD	*10	c.2983G>T	p.V995F	rs1801268
101	DPYD	*2A	IVS14+1G>A	-	rs3918290
102	DPYD	*7	c.295-298delTCAT	-	rs72549309
103	DPYD	*8	-	p.R235W	rs1801266
104	DPYD	*9A	c.85T>C	p.C29R	rs1801265
105	DPYD	*9B	c.2657G>A	p.R886H	rs1801267
106	GSTM1	*B	c.519G>C	p.K172N	rs1065411
107	GSTM1	CNV	-	-	-
108	GSTP1	*A_*B_*C_*D	c.313A>G	p.I105V	rs1695
109	GSTP1	*A_*B_*C_*D	c.341C>T	p.A114V	rs1138272
110	GSTT1	CNV	-	-	-
111	NAT1	*11	c.-344C>T	-	rs4986988
112	NAT1	*11	c.-40A>T	-	rs4986989
113	NAT1	*11	c.445G>A	p.V149I	rs4987076
114	NAT1	*11	c.459G>A	p.T153T	rs4986990
115	NAT1	*11	c.640T>G	p.S214A	rs4986783
116	NAT1	*14	c.560G>A	p.R187Q	rs4986782
117	NAT1	*15	c.559C>T	p.R187X	rs5030839
118	NAT1	*17	c.190C>T	p.R64W	rs56379106
119	NAT1	*19	c.97C>T	p.R33X	rs56318881
120	NAT1	*22	c.752A>T	p.D251V	rs56172717
121	NAT1	*5(350-351G>C)	c.350-351GG>CC	p.R117T	rs72554606

122	NAT1	*5(497-499G>C)	c.497-499GGG>CCC	p.R166T; E167Q	rs72554608
123	NAT1	*5(884A>G)	c.884A>G	-	rs55793712
124	NAT1	*5(976delA)	c.976delA	-	rs72554612
125	NAT2	*11	c.481C>T	p.L161L	rs1799929
126	NAT2	*12	c.803A>G	p.K268R	rs1208
127	NAT2	*13	c.282C>T	p.Y94Y	rs1041983
128	NAT2	*14	c.191G>A	p.R64Q	rs1801279
129	NAT2	*19	c.190C>T	p.R64W	rs1805158
130	NAT2	*5	c.341T>C	p.I114T	rs1801280
131	NAT2	*6	c.590G>A	p.R197Q	rs1799930
132	NAT2	*7	c.857G>A	p.G286E	rs1799931
133	SLC15A2	A284A	c.852G>A	p.A284A	rs2293616
134	SLC15A2	L350F	c.1048C>T	p.L350F	rs2257212
135	SLC15A2	P409S	c.1225C>T	p.P409S	rs1143671
136	SLC15A2	R509K	c.1526G>A	p.R509K	rs1143672
137	SLC22A1	420del	c.1258-1260delATG	p.M420del	rs35167514; rs34305973; rs35191146
138	SLC22A1	C88R	c.262T>C	p.C88R	rs55918055
139	SLC22A1	G220V	c.659G>T	p.G220V	rs36103319
140	SLC22A1	G401S	c.1201G>A	p.G401S	rs34130495
141	SLC22A1	G465R	c.1393G>A	p.G465R	rs34059508
142	SLC22A1	M408V	c.1222A>G	p.M408V	rs628031
143	SLC22A1	P283L	c.848C>T	p.P283L	rs4646277
144	SLC22A1	P341L	c.1022C>T	p.P341L	rs2282143
145	SLC22A1	R287G	c.859C>G	p.R287G	rs4646278
146	SLC22A1	R61C	c.181C>T	p.R61C	rs12208357
147	SLC22A2	K432Q	c.1294A>C	p.K432Q	rs8177517
148	SLC22A2	M165I	c.495G>A	p.M165I	rs8177507
149	SLC22A2	P54S	c.160C>T	p.P54S	rs8177504
150	SLC22A2	R400C (*7.1)	c.1198C>T	p.R400C	rs8177516
151	SLC22A2	S270A (*4.1)	c.808G>T	p.S270A	rs316019
152	SLC22A6	R50H	c.149G>A	p.R50H	rs11568626
153	SLCO1B1	*10	c.1964A>G	p.D655G	rs56199088
154	SLCO1B1	*11	c.2000A>G	p.E667G	rs55737008
155	SLCO1B1	*12			
156	SLCO1B1	*13			
157	SLCO1B1	*1B	c.388G>A	p.N130D	rs2306283
158	SLCO1B1	*2	c.217T>C	p.F73L	rs56101265
159	SLCO1B1	*3(E156G)	c.467A>G	p.E156G	rs72559745
160	SLCO1B1	*3(V82A)	c.245T>C	p.V82A	rs56061388
161	SLCO1B1	*5	c.521T>C	p.V174A	rs4149056

162	SLCO1B1	*6	c.1058T>C	p.I353T	rs55901008
163	SLCO1B1	*9	c.1463G>C	p.G488A	rs59502379
164	SLCO2B1	*3	c.1457C>T	p.S486F	rs2306168
165	SLCO1B3	M233I	c.699G>A	p.M233I	rs7311358
166	SLCO1B3	S112A	c.334T>G	p.S112A	rs4149117
167	SULT1A1	*2	c.638G>A	p.R213H	rs9282861
168	SULT1A1	*3(M223V)	c.667G>A	p.V223M	rs1801030
169	SULT1A1	*4(R37Q)		p.R37Q	rs72547527
170	SULT1A1	Null(deletion)			NA
171	TPMT	*2	c.238G>C	p.A80P	rs1800462
172	TPMT	*3a			
173	TPMT	*3b	c.460G>A	p.A154T	rs1800460
174	TPMT	*3c	c.719A>G	p.Y240C	rs1142345
175	TPMT	*8	c.644G>A	p.R215H	rs56161402
176	UGT1A1	*27	c.686C>A	p.P229Q	rs35350960
177	UGT1A1	*29	c.1099C>G	p.R367G	rs55750087
178	UGT1A1	*6	c.211G>A	p.G71R	rs4148323
179	UGT1A1	*60	-3279T>G	-	rs4124874
180	UGT1A1	*7	c.1456T>G	p.Y486D	rs34993780
181	UGT2B15	*2	c.253G>T	p.D85Y	rs1902023
182	UGT2B17	CNV			NA
183	UGT2B7	*2	c.802T>C	p.H268Y	rs7439366
184	VKORC1	-1639G>A	-1639G>A	-	rs9923231

Supplement Table 2. The demographic data and PASI of the patients in two phases

Phase/ Genotype	Effective Group	Age		BMI		PASI baseline	
		Value	P value	Value	P value	Value	P value
Discovery phase	PASI<50(23)	45.35±14.06	0.066	23.11±3.90	0.936	10.41±7.08	0.477
	PASI>50(23)	36.95±16.03		23.18±2.29		12.08±8.57	
Verification phase	PASI<50(57)	42.45±14.00	**0.013	22.73±5.19	0.609	10.73±7.41	0.671
	PASI>50(48)	35.30±15.67		22.32±2.49		11.28±6.21	
SLCO1B1 rs4149056 TT		38.68±12.14	0.369	22.91±2.41	0.466	11.17±4.84	0.381
SLCO1B1 rs4149056TC+CC		39.76±14.82		22.77±3.49		11.20±4.10	
SLC22A1 rs2282143 CC		40.41±13.64	0.576	22.72±3.63	0.468	12.24±5.24	0.128
SLC22A1 rs2282143CT+TT		40.39±11.25		22.90±2.52		10.23±4.12	

Supplement Table 3. The polymorphisms of rs4149056T>C and rs2282143C>T frequencies in patients with PASI>10

phases	SLC22A1 Rs2282143						SLCO1B1 Rs4149056					
	Genotypes/alleles	PASI<50	PASI>50	P value	OR[95CI]	HWE	Genotypes/alleles	PASI<50	PASI>50	P value	OR[95CI]	HW
Discovery Phase	CC	12	3	P<0.001	40.000[3.579, 447.034]	0.171	TT	12	6	P=0.011	14.000[1.385, 141.485]	0.354
	CT+TT	1	10				TC	1	7			
	C	25	16	P=0.002	15.625[1.821,134.040]		T	25	19	P=0.021	9.211[1.043,81.361]	
	T	1	10				C	1	7			
Verification Phase	CC	28	19	P=0.012	5.404[1.328, 21.983]	0.311	TT	29	24	P=0.117	3.625[0.669, 19.630]	0.584
	CT+TT	3	11				TC	2	6			
	C	59	49	P=0.019	4.415[1.166, 16.720]		T	60	54	P=0.131	3.333[0.645,17.218]	
	T	3	11				C	2	6			
Total samples	CC	40	22	P<0.001	9.545[2.907, 31.348]	0.118	TT	41	30	P=0.005	5.922[1.549, 22.635]	0.345
	CT+TT	4	21				TC+TT	3	13			
	C	84	65	P<0.001	6.785[2.220, 20.736]		T	85	73	P=0.008	5.046[1.384, 18.399]	
	T	4	21				C	3	13			

Supplement Table 4. The haplotypes of rs2282143-rs4149056 polymorphisms distribution in response and non-response groups.

Haplotypes (rs2282143-rs4149056)	Response (N, %)	Non-response (N, %)	Sig.
CC-TT	32(45.07%)	63(78.75%)	P<0.001
CT-TT/CC-CT	21(29.58%)	14(17.5%)	
CT-CT	18(25.35%)	3(3.75%)	
CC-TT/CT-TT/CC-CT (<2 variants)	53(74.65%)	77(96.25%)	P<0.001
CT-CT	18(25.35%)	3(3.75%)	
